# A Boolean Model of Microvascular Rarefaction to Predict Treatment Outcomes in Renal Disease

**DOI:** 10.1038/s41598-019-57386-8

**Published:** 2020-01-16

**Authors:** Erika Williams, Alejandro R. Chade

**Affiliations:** 1 The Department of Physiology and Biophysics, University of Mississippi Medical Center, Jackson, MS USA; 2 The Department of Medicine, University of Mississippi Medical Center, Jackson, MS USA; 3 The Department of Radiology, University of Mississippi Medical Center, Jackson, MS USA

**Keywords:** Glomerulus, Renal artery stenosis

## Abstract

Despite advances in renovascular disease (RVD) research, gaps remain between experimental and clinical outcomes, translation of results, and the understanding of pathophysiological mechanisms. A predictive tool to indicate support (or lack of) for biological findings may aid clinical translation of therapies. We created a Boolean model of RVD and hypothesized that it would predict outcomes observed in our previous studies using a translational swine model of RVD. Our studies have focused on developing treatments to halt renal microvascular (MV) rarefaction in RVD, a major feature of renal injury. A network topology of 20 factors involved in renal MV rarefaction that allowed simulation of 5 previously tested treatments was created. Each factor was assigned a function based upon its interactions with other variables and assumed to be “on” or “off”. Simulations of interventions were performed until outcomes reached a steady state and analyzed to determine pathological processes that were activated, inactivated, or unchanged vs. RVD with no intervention. Boolean simulations mimicked the results of our previous studies, confirming the importance of MV integrity on treatment outcomes in RVD. Furthermore, our study supports the potential application of a mathematical tool to predict therapeutic feasibility, which may guide the design of future studies for RVD.

## Introduction

Major advances in nephrology research have been achieved thanks to numerous experimental studies that have elucidated underlying mechanisms of deteriorating renal function and identified therapeutic targets with potential for clinical translation. Nevertheless, the transition to clinical settings is usually slower than expected or, often, exciting findings from experimental platforms are not reproduced in patients^1–3^. Thus, attempts at addressing how all of these pathophysiological mechanisms may interact and function simultaneously in disease states could contribute to the predictive quality of experimental findings.

A Boolean model is a type of discrete modeling that describes qualitative aspects of a network to convert background knowledge of a biological system into a computable algorithm. Although Boolean models, like continuous mathematical models, are considered quantitative, Boolean models have a qualitative nature in the fact that they do not predict specific values for each variable, but rather whether each variable is active or inactive at any given point in time. Boolean models are often less complex than continuous mathematical models and use networks to determine the state of each variable involved in the network. By assuming that each component of the network is always either activated or inhibited based on its interactions with other variables, the steady state of the system can be determined. The use of Boolean networks in mathematical modeling has several advantages, including their intuitive nature, ease of parameterization compared to models given as systems of differential equations, the ability to derive predictions of qualitative behavior of a system, and ease of confirming outcomes experimentally^4^.

**Figure 1:**
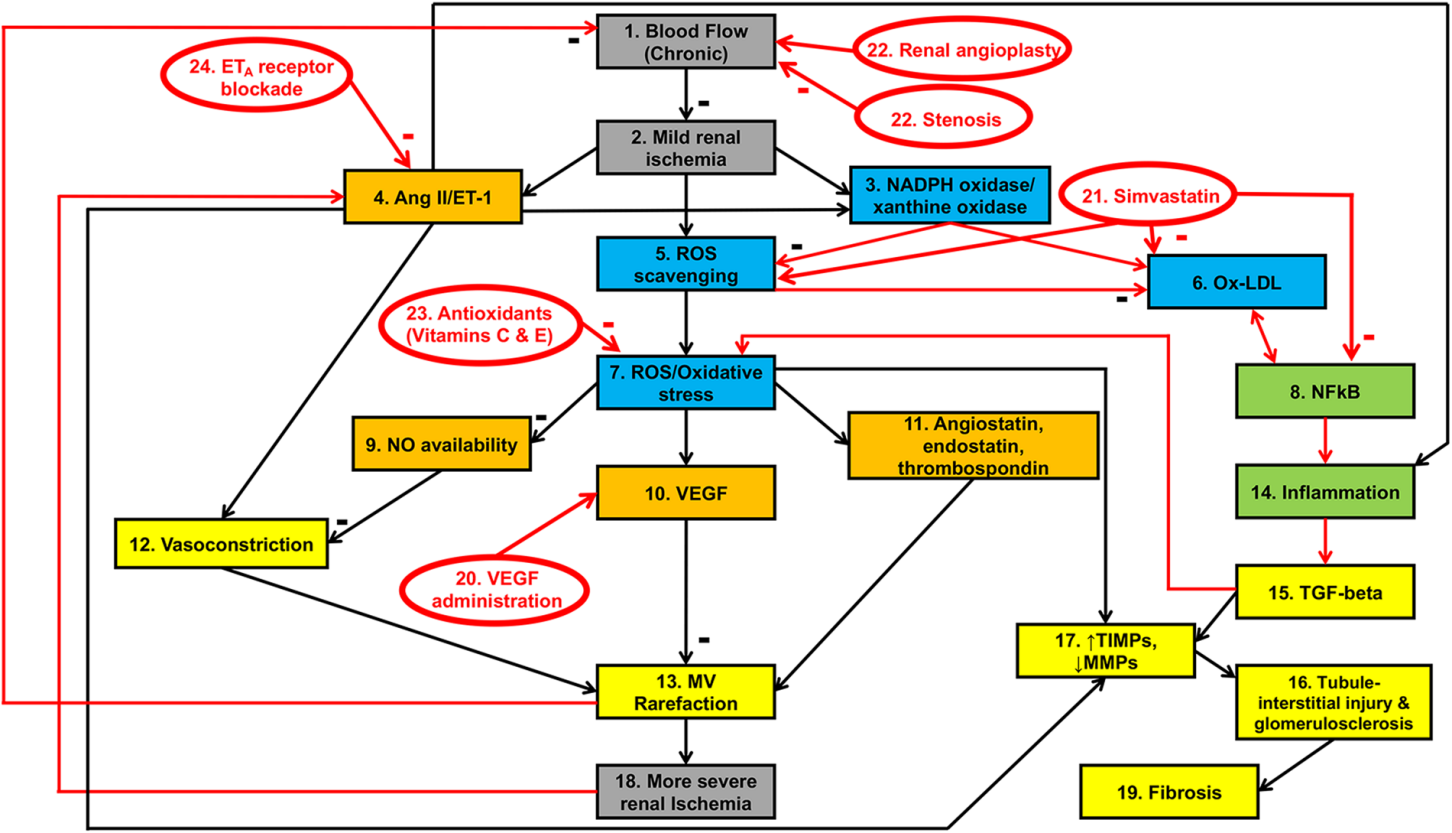
Network topology framework of MV rarefaction in RVD: Schematic illustration of the mechanisms involved in MV rarefaction and renal injury in RVD. Black arrows indicate a temporal relationship between variables. Red arrows indicate feed-forward cycles between variables. Variables are grouped to indicate the pathophysiological process they are most directly related to. Gray factors relate to oxygenation of the kidney and development (and progression) of renal ischemia; orange factors relate to MV endothelial function and integrity; blue factors relate to oxidative stress; green factors relate to inflammation; major outcomes of interest (reflecting pathophysiological consequences) are depicted in yellow.

A major research focus of our laboratory is elucidating pathophysiological mechanisms of chronic renovascular disease (RVD) and the development of new therapies to recover renal function. Renal MV rarefaction is a major pathological feature of chronic renal diseases independent of the etiology^5,6^ and associates with progression of renal injury^7,8^. Our previous studies using a swine model of chronic RVD showed that renal MV rarefaction develops and progresses along with deteriorating renal function, paired with blunted renal MV repair and increased MV remodeling. These processes associate with and are likely driven by decreased renal bioavailability of vascular endothelial growth factor (VEGF) and altered downstream angiogenic signaling^9–11^, since proof-of-concept studies showed that preventive^8^ or interventional^12,13^ intra-renal administration of VEGF successfully improved renal function and preserved MV integrity while reducing renal injury^7,8^ in RVD. Furthermore, we showed that inhibition of factors involved in oxidative stress, inflammation, and vasoconstriction, and stimulation of angiogenic signaling in RVD improved stenotic kidney hemodynamics, fibrosis, and associates with preserved MV architecture (Fig. 1)^7,8,12–20^. Such findings not only support the importance of the renal MV integrity for renal function in RVD, but also suggest a network of numerous factors that are part of a vicious cycle driving progressive MV rarefaction that may ultimately contribute to the declining renal function.

A predictive tool to test and indicate support (or lack of) for our biological findings would be of utmost importance not only to advance towards clinical applications but may also help with more efficient design of future studies. To our knowledge, an integrative Boolean model of MV rarefaction in RVD has not yet been described. We designed a Boolean model that integrates the complex pathophysiology of MV rarefaction and r enal deterioration for a comprehensive description of the disease process and responses to experimental therapies. Our goal is multifold: A) to define the predictive quality of the current understanding of renal pathophysiology in RVD, and B) the potential of our experimental therapies in RVD to translate into clinical practice. We hypothesize that the Boolean model of MV rarefaction will predict outcomes observed in our published studies using the swine model of RVD.

## Results

It should be noted that, for this Boolean model of MV rarefaction in RVD, we are interested in simulating the outcomes of therapies previously tested only in our experimental swine model of RVD. However, the composition of the Boolean model in the current study as well as the pathophysiological traits of MV rarefaction in renal disease are supported by previous work using various models of renal disease^21–28^. It is also important to note that the determination of whether a variable is considered activated (“on”) or inactivated (“off”) is based upon a range dictated by the referenced previously published studies in which each value was measured experimentally. In many cases, when a variable is predicted to be inactivated by the Boolean model, this does not translate to a physiologic complete lack or inactivity of the variable, as many of these factors are constitutively expressed or activated in any given state. The experimental measurements quantified and used to determine the status of each variable included in the model are listed in Table 1.

**Table 1:** Variables included in the Boolean model and experimental measurements used as comparison.

Variable	Experimental Measurement
Chronic blood flow	CT-derived renal blood flow
Mild renal ischemia	BOLD MRI^67,68^ Renal HIF-1α expression
NADPH Oxidase/Xanthine Oxidase	Renal p47phox, p67phox, gp91, and xanthine oxidase expression
Ang II/ET-1	Plasma renin activity Blood and urine ET-1 concentration
ROS Scavenging	Renal superoxide dismutase activity
Ox-LDL	Plasma Ox-LDL. Renal expression of Ox-LDL receptor
ROS/Oxidative Stress	Renal superoxide anion and nitrotyrosine expression Renal superoxide dismutase activity
NFkB	Renal NFkB expression and activity
Nitric Oxide	Renal p-eNOS expression
VEGF	Renal VEGF expression and availability
Angiostatin/Endostatin/Thrombospondin	Renal angiostatin, endostatin, and thrombospondin expression
Vasoconstriction	Renal responses to vasoactive challenge (intra-renal acetylcholine and sodium nitroprusside)
MV rarefaction	Renal MV density
Inflammation	Renal iNOS and MCP-1 expression Renal infiltrates of inflammatory cells
TGF-beta	Renal TGF-beta expression
Tubule-interstitial injury and glomerulosclerosis	Area of fibrotic tissue (%) Glomerular score
TIMPS/MMPs	Renal TIMP-1 and MMP-2 and -9 expression
More severe renal ischemia	BOLD MRI^67,68^ Renal HIF-1α expression
Fibrosis	Area of fibrotic tissue (%) Renal tTG and CTGF expression

The experimental measurement to quantify each variable included in the model is listed next to the variable name. After measurement of each variable, determination as to what constitutes each variable being either “on” or “off” is based upon the measured value in normal non-RVD pigs. BOLD MRI, blood oxygen level-dependent magnetic resonance imaging; HIF-1α, hypoxia-inducible factor 1α; p47phox, neutrophil cytosolic factor 1; p67phox, neutrophil cytosolic factor 2; gp91, NADPH oxidase 2; p-eNOS, phosphorylated endothelial nitric oxide synthase; iNOS, inducible nitric oxide synthase; tTg, tissue transglutaminase; CTGF, connective tissue growth factor.

### Initial Boolean model simulation of RVD

Each simulation of each therapeutic intervention began with the same simulation of RVD by activating renal artery stenosis, which chronically inhibits blood flow in the Boolean model.

### Boolean model of RVD reproduces the outcomes observed in human and swine RVD

**Figure 2:**
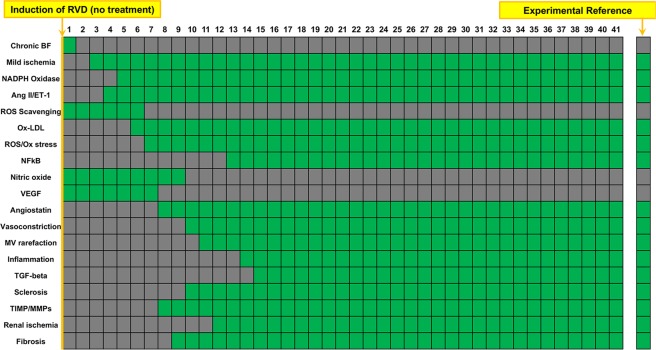
Simulation of RVD with no therapeutic intervention: Simulation of RVD by chronically inactivating blood flow to the kidney results in activation of multiple factors involved in oxidative stress, inflammation, MV rarefaction, and fibrosis, all processes that contribute to the progression of renal function deterioration *in vivo*. Names of the factors are identified in the y axis and indicated in green when active and are depicted in gray when inactive. Numbers on the x axis depict the arbitrary timepoints or “cycles” run by the simulation. The first column depicts the initial steady state condition at which time the simulation has been initiated but has not yet produced changes.

As shown in Fig. 2, prolonged renal artery stenosis leads to a progressive activation of deleterious processes in the kidney, ending in a steady state in which vasoconstriction, mild ischemia, variables involved in oxidative stress, inflammation, and fibrosis were activated. Simultaneously, “protective” variables including VEGF, NO, and scavenging of ROS were inactivated in the steady state. Overall, this profile of increased MV rarefaction is in line with what we have observed experimentally in the swine model of RVD^7–9,29^.

### Boolean simulation of RVD with simvastatin administration reproduces the improved outcomes of the swine model

**Figure 3:**
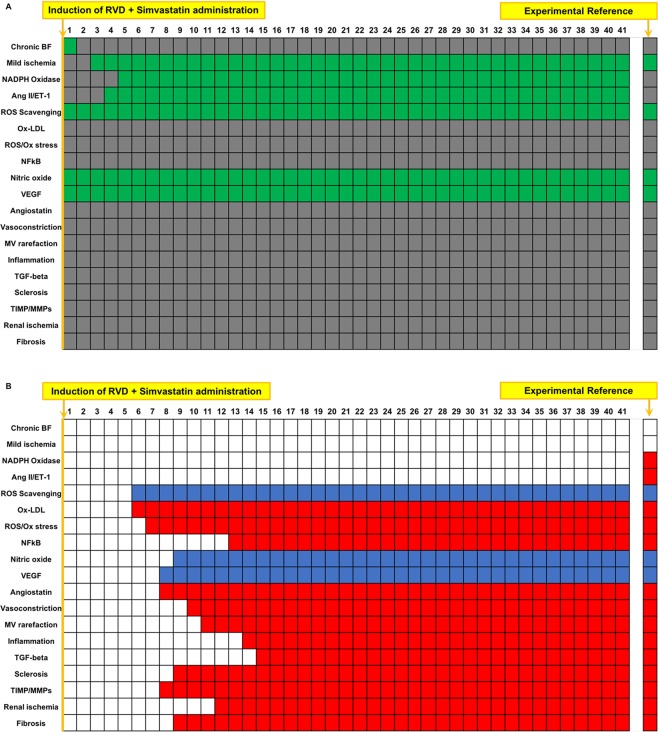
Simulation of RVD treated with simvastatin: Simulation of RVD with simvastatin therapy results in a deactivation of factors involved in oxidative stress, inflammation, fibrosis, and MV rarefaction compared to simulation of RVD with no treatment, while increasing ROS scavenging and factors involved in angiogenic signaling and improved endothelial function, including increased VEGF and NO with a simultaneous reduction in angiostatin, vasoconstriction, and MV rarefaction/regression. Importantly, simvastatin treatment was predicted to be unable to inactivate tissue hypoxia, Ang II, or NADPH oxidase. Names of the factors are identified in the y axis and indicated in green when active and are depicted in gray when inactive (**A**). Numbers on the x axis depict the arbitrary timepoints or “cycles” run by the simulation. Variables that were inactive after simvastatin simulation compared to untreated RVD control are depicted in red, and factors that activated are depicted in blue, with the x axis demonstrating at which timepoint this switch occurred (**B**). The first column in (**A,B**) depicts the initial steady state condition at which time the simulation has been initiated but has not yet produced changes.

Statin treatment was simulated at timepoint 1, which activates ROS scavenging while inhibiting Ox-LDL and NFkB^14,30^. Examination of the steady state reached by the simulation reveals that simvastatin treatment during RVD halts many deleterious processes in the kidney (Fig. 3A) compared to untreated RVD, including vasoconstriction, factors associated with inflammation and fibrosis, and overall inactivation of MV rarefaction and regression. Deleterious variables that remain active or unchanged compared to untreated RVD in the steady state include mild ischemia, Ang II/ET-1, NADPH oxidase, and oxidative stress. However, the balance between activated injurious processes and activated protective processes led to favorable endpoint outcomes in the simulation that are in line with what we observed experimentally^14,30^, including attenuated MV rarefaction, fibrosis, and glomerulosclerosis in the steady state. This can be further appreciated in Fig. 3B, which conveys that whereas ROS scavenging, NO, and VEGF were activated after Simvastatin administration compared to untreated RVD, each deleterious variable was either inactivated or unchanged compared to untreated RVD. This finding both predicts and confirms previous results that targeting oxidative stress therapeutically may be sufficient to beneficially alter important downstream pathophysiological processes in the stenotic kidney but does not ameliorate several key upstream alterations^14,30^.

### Boolean simulation of RVD with anti-oxidant administration reproduces the improved outcomes of the swine model

**Figure 4:**
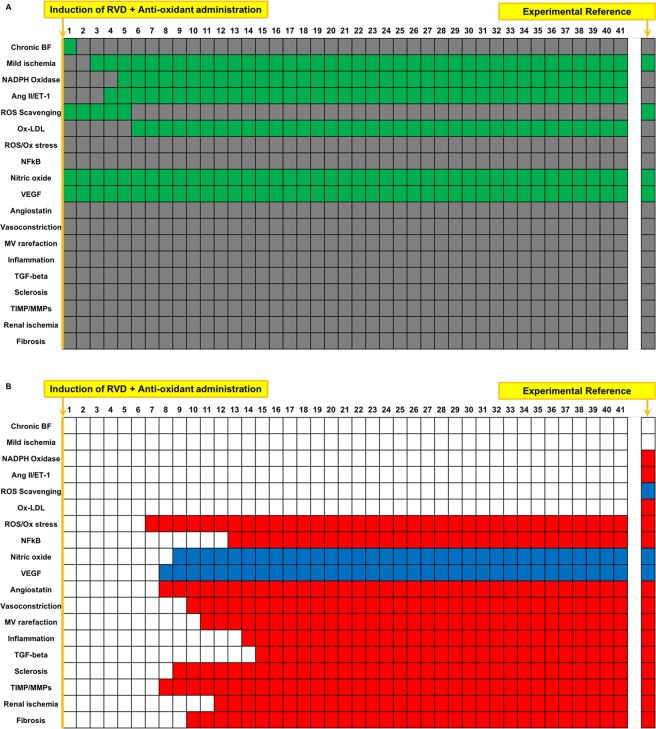
Simulation of RVD treated with anti-oxidant (Vitamins C and E): Simulation of RVD with anti-oxidant administration results in a decrease in factors involved in oxidative stress, inflammation, fibrosis, and MV rarefaction compared to simulation of RVD with no treatment, while increasing factors involved in angiogenic signaling and improved endothelial function. Despite an overall beneficial profile, anti-oxidant administration was unable to combat mild ischemia, NADPH oxidase, Ang II, ROS scavenging, or Ox-LDL, though there was no change in activity of these variables. Names of the factors are identified in the y axis and indicated in green when active and are depicted in gray when inactive (**A**). Variables that were inactive after anti-oxidant administration simulation compared to untreated RVD control are depicted in red, and factors that activated are depicted in blue (**B**). The first column in (**A,B**) depicts the initial steady state condition at which time the simulation has been initiated but has not yet produced changes.

Anti-oxidant treatment was simulated at timepoint 1, which inactivates ROS/oxidative stress^15–17^. Similar results as simulation of treatment with Simvastatin were observed (Fig. 4A), including inactivated MV rarefaction, fibrosis, and glomerulosclerosis, confirming experimental observations^15–17^. Interestingly, unlike with simvastatin treatment, Ox-LDL was activated (unchanged compared to untreated RVD, Fig. 4B) while ROS scavenging was inactivated. Overall, compared to untreated RVD, simulation of anti-oxidant administration resulted in activation of NO and VEGF with inactivation of MV rarefaction, fibrosis, and glomerulosclerosis (Fig. 4B). Similar to simvastatin therapy, the Boolean model once again both predicts and confirms experimental observations that sole targeting of oxidative stress may leave some pathophysi ological mechanisms unaltered, potentially allowing further progression of the disease.

### Boolean simulation of RVD with ET_A_ receptor blockade reproduces the improved outcomes of the swine model

**Figure 5:**
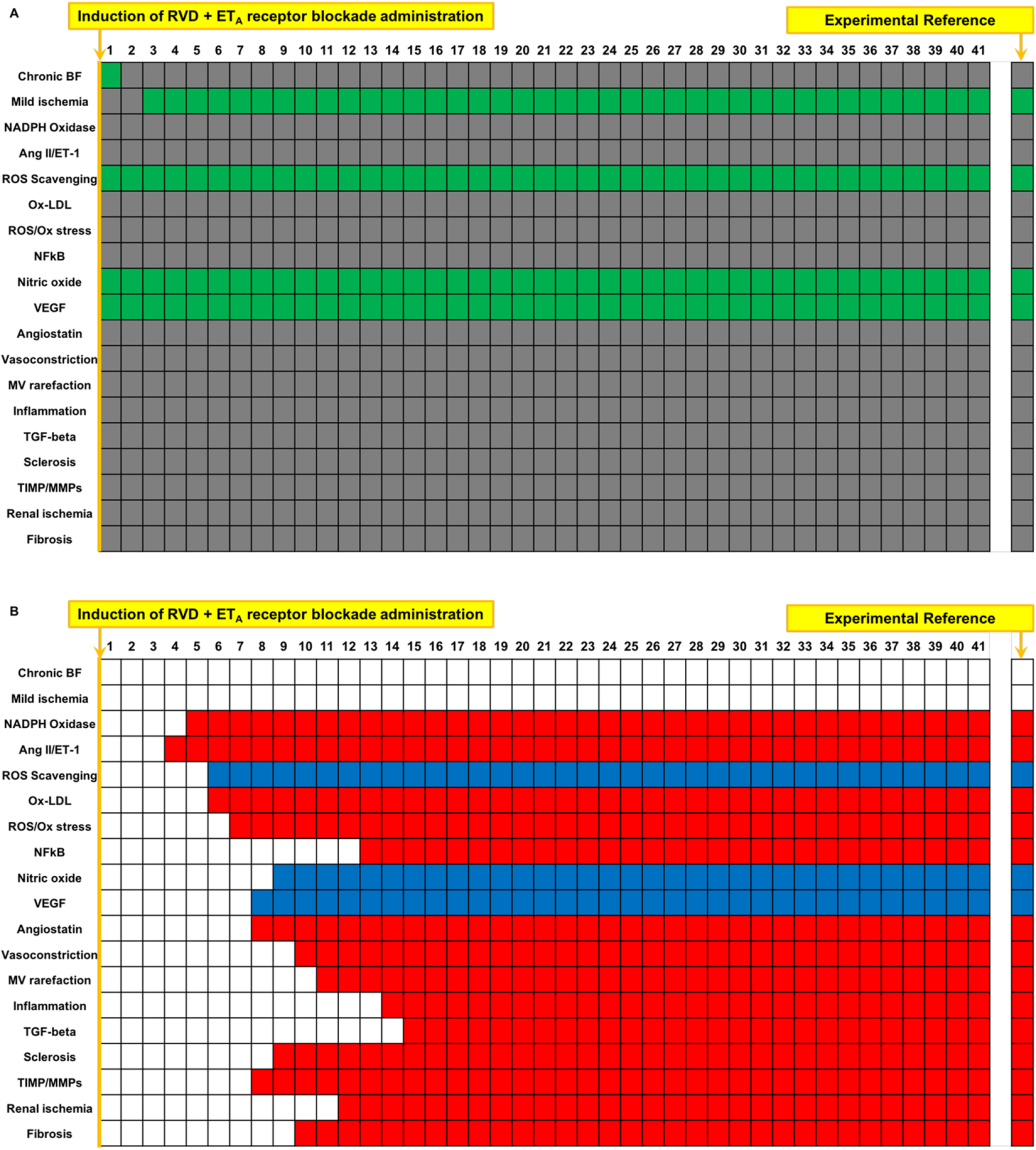
Simulation of RVD treated with ET_A_ receptor blockade: Simulation of RVD with ET_A_ receptor blockade results in a decrease in factors involved in oxidative stress, inflammation, fibrosis, and MV rarefaction compared to simulation of RVD with no treatment, while increasing reactive oxygen species scavenging and factors involved in angiogenic signaling and improved endothelial function. As with other targeted treatments that did not involve direct restoration of blood flow to the kidney, chronic blood flow and mild ischemia remained unchanged after ET_A_ blockade simulation. Names of the factors are identified in the y axis and indicated in green when active and are depicted in gray when inactive (**A**). Variables that were inactive after ET_A_ blockade simulation compared to untreated RVD control are depicted in red, and factors that activated are depicted in blue (**B**). The first column in (**A,B**) depicts the initial steady state condition at which time the simulation has been initiated but has not yet produced changes.

ET_A_ receptor blockade treatment was simulated at timepoint 1, interfering with the Ang II/ET-1 axis^18,19,31^. Similar to experimental observations^18,19,31^, ET_A_ receptor blockade improved MV rarefaction, inflammation, oxidative stress, and fibrosis, while preserving VEGF and NO compared to untreated RVD, mimicking the protective effects of ET_A_ receptor blockade in experimental RVD (Fig. 5A). As shown in Fig. 5B, the Boolean model produced end steady states for each variable that precisely matched our expected outcomes based on experimental references after RVD with ET_A_ receptor blockade simulation compared to untreated RVD.

### Boolean simulation of RVD with preventive VEGF therapy closely reproduces the improved outcomes of the swine model

**Figure 6:**
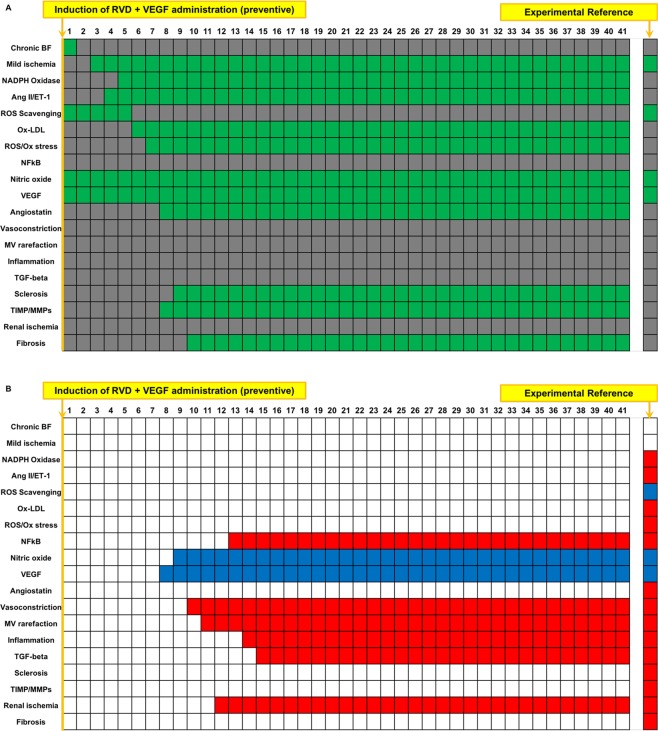
Simulation of RVD treated with a single administration of VEGF (preventive): Simulation of RVD with VEGF administration performed at induction of RVD results in a decrease in factors involved in inflammation and MV rarefaction compared to RVD with no treatment, with an increase in variables involved in angiogenesis and improved endothelial function. Several variables involved in oxidative stress, fibrosis, and glomerulosclerosis remained unchanged. Names of the factors are identified in the y axis and indicated in green when active and are depicted in gray when inactive (**A**). Variables that were inactive after preventative VEGF administration simulation compared to untreated RVD control are depicted in red, and factors that activated are depicted in blue (**B**). The first column in (**A,B**) depicts the initial steady state condition at which time the simulation has been initiated but has not yet produced changes.

VEGF was simulated by activating VEGF administration at timepoint 1, which activates VEGF in the network^7,10^. As observed experimentally^7^, VEGF at the onset of RVD inactivated MV rarefaction and regression, along with variables involved in inflammation and oxidative stress compared to untreated RVD (Fig. 6). Interestingly, glomerulosclerosis and fibrosis were activated in the steady state of this simulation (unchanged vs. untreated RVD, Fig. 6B), which was unexpected based upon observed experimental outcomes. However, it should be noted that TGF-beta, NFkB, and inflammation, which directly impact fibrosis and glomerulosclerosis, are inactivated in the Boolean simulation of RVD with VEGF therapy. Therefore, it is possible that, although fibrosis and glomerulosclerosis are activated in the steady state in the model, the deactivation of factors affecting these disease states could reflect their attenuation when examined experimentally *in vivo*, as we showed^7,10^.

### Boolean simulation of RVD with interventional VEGF therapy reproduces the improved outcomes of the swine model

**Figure 7:**
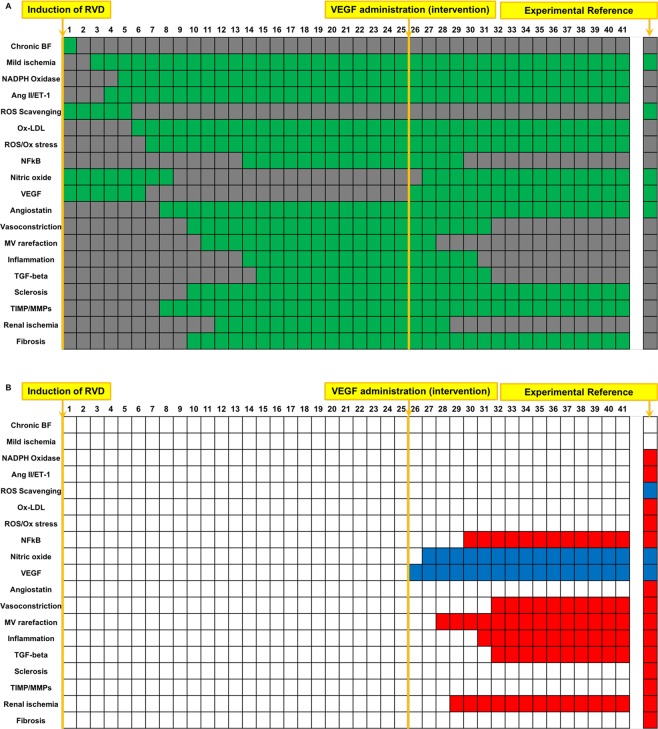
Simulation of RVD treated with a single administration of VEGF (interventional): In comparison to VEGF administration given at induction of RVD, VEGF administered after progression of RVD results in a decrease in factors involved in inflammation and oxidative stress, with increases in variables involved in angiogenesis and improved endothelial function. Interestingly, variables involved in oxidative stress, fibrosis, and glomerulosclerosis remained activated following interventional VEGF therapy. Names of the factors are identified in the y axis and indicated in green when active and are depicted in gray when inactive (**A**). Variables that were inactive after interventional VEGF administration simulation compared to untreated RVD control are depicted in red, and factors that activated are depicted in blue (**B**). VEGF therapy simulation began at timepoint 25. The first column in (**A,B**) depicts the initial steady state condition at which time the simulation has been initiated but has not yet produced changes.

VEGF therapy was simulated by activating VEGF administration at timepoint 25, rather than at timepoint 1^8,12,20^. The simulation predicted very similar outcomes as the simulation of VEGF administered at the onset of disease (Fig. 7), including activation and inactivation of the same variables compared to untreated RVD (Fig. 7B**)** indicating similar efficacy on MV and renal protection when VEGF is given as an interventional or as a preventive treatment^8,12,20^.

### Boolean simulation of RVD with co-adjuvant VEGF therapy to renal angioplasty reproduces the improved outcomes of the swine model

**Figure 8:**
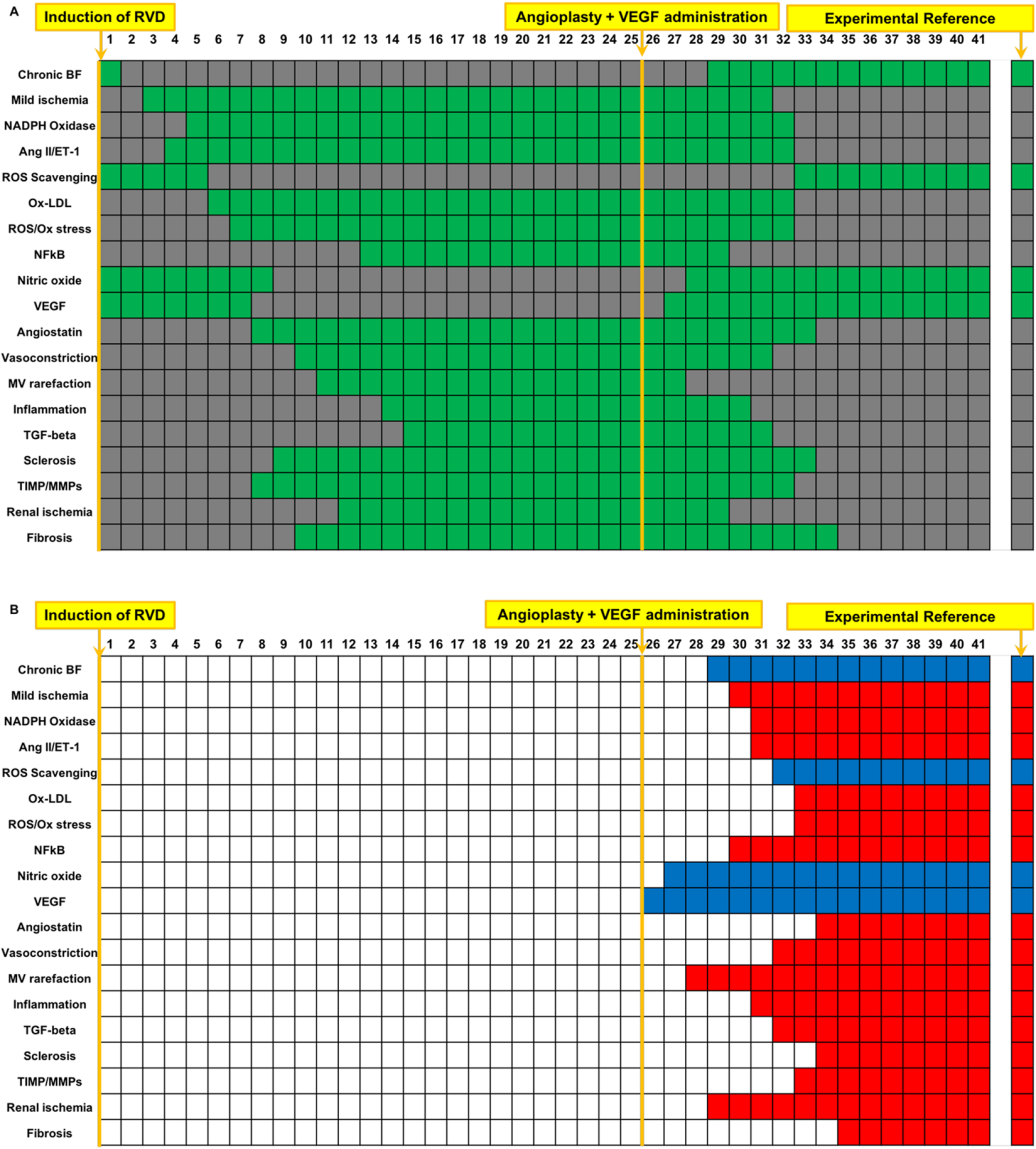
Simulation of RVD treated with co-adjuvant renal angioplasty and VEGF administration: Simulation of RVD with simultaneous angioplasty and co-adjuvant VEGF administration demonstrated an initial increase in variables involved in deleterious processes associated with renal injury progression prior to therapy, with a subsequent reversal following the combined therapy. The end state closely mimicked the state of the healthy kidney with inactive inflammation, MV rarefaction, fibrosis, and glomerulosclerosis, and activated angiogenesis and scavenging of reactive oxygen species. The impact of the treatment simulation to either inactivate or activate every single variable in the network, which did not occur in any other simulation, is likely due to the direct restoration of blood flow to the stenotic kidney, which allowed an inactivation of mild ischemia, Ang II, and NADPH oxidase. Names of the factors are identified in the y axis and indicated in green when active and are depicted in gray when inactive (**A**). Variables that were inactive after co-adjuvant renal angioplasty and VEGF administration simulation compared to untreated RVD control are depicted in red, and factors that activated are depicted in blue (**B**). Renal angioplasty and VEGF therapy simulation began at timepoint 25. The first column in (**A,B**) depicts the initial steady state condition at which time the simulation has been initiated but has not yet produced changes.

Renal angioplasty was simulated by inactivating stenosis at timepoint 25, and simultaneously VEGF therapy was simulated by activating exogenous VEGF at timepoint 25^13^. The simulation yielded very positive outcomes that mimicked those observed recently in the swine RVD model^7,13^, including inactivation of MV rarefaction and regression, fibrosis, glomerulosclerosis, inflammation, oxidative stress, and factors involved in these processes compared to untreated RVD (Fig. 8). At the same time, this multi-targeted therapeutic approach successfully recovered and activated VEGF, NO, and ROS scavenging. Importantly, the endpoint outcomes predicted by the Boolean model confirms our recent work^13^ that supports the notion that this combined strategy may be superior to targeting a single pathophysiological factor.

## Discussion

Our study supports a novel application of a relatively simple mathematical tool that could be used to indicate support for and possibly predict biological findings and estimate overall outcomes of RVD with and without therapeutic interventions. Based upon known pathophysiology of RVD and the prominent role that MV disease plays on the development and progression of renal injury, we developed a Boolean model of MV rarefaction and confirmed its predicted outcomes against tested therapeutic strategies in our swine model of RVD. The Boolean model successfully predicted the observed outcomes of experimental studies using a breadth of different therapeutic interventions^7,8,12–17,19,20,30–32^. Furthermore, and perhaps of higher importance based on recent clinical data^33,34^, the model predicted that a combined strategy of renal angioplasty with VEGF therapy is more effective at recovering stenotic kidney hemodynamics and function than renal angioplasty alone, which reproduced our recent findings^13^ and may open new avenues for therapeutic developments. Our experimental observations coupled with confirmation by the Boolean model highlight and confirm the importance of renal MV integrity on the progression of renal injury in RVD and recovery after treatments. Thus, the predictive quality of this mathematical tool may contribute to define whether or not clinical translation of experimental therapies might be feasible and, possibly, reproducible.

Animal models offer unique experimental platforms to understand pathophysiology and to test (known or experimental) therapies. The swine model of RVD is a great model to study cardiovascular and renal disease in a translational fashion^35,36^ and to test therapeutic interventions targeting factors involved in th e development and progression of MV rarefaction, such as inhibition of the RAAS and endothelin pathways^7,8,12,13,18–20^, oxidative stress^15,17^, and inflammation^14^. We showed that those treatments induced significant amelioration of renal injury and were possible to mimic with the Boolean model, supporting the attractive notion that general outcomes of experimental therapies may not only be confirmed, but also, to some extent, might be predicted by using this tool before launching *in vivo* (experimental or clinical) and possibly *in vitro* studies.

Although the current study is based upon application of the Boolean model with studies using a swine model of RVD, this issue does not rule out the potential application to other representative experimental platforms of renal disease. Renal MV rarefaction is not exclusive to RVD or the swine RVD model. In fact, MV rarefaction has been observed in several renal pathologies and is a universal feature in acute and chronic renal disease irrespective of the etiology or platform^5,6^. Thus, it is possible that our mathematical approach may be applied and used as an early predictive tool in other models of renal disease, especially given the potential for easy modifications. Novel therapeutic interventions may be relatively easily added to the Boolean model as well, as long as its target plays a known role in MV rarefaction. A unique benefit of the Boolean model is that rules may be added to include new therapeutic interventions or biological variables as discovered without the need to re-work the entire network.

Whereas our animal studies are designed to emulate a chronic and often progressive condition observed in patients, the Boolean model is limited by the fact that it cannot mimic the specific lengths of time that we follow in the swine RVD model. Rather, we are only able to model an arbitrary timescale that does not match up with “real time”. To address this potential limitation on the timescale in which the model runs and in order to simulate MV rarefaction in RVD as closely as we can, we instead allow the simulation to run until each variable has reached a steady state and does not change for multiple time cycles. This still closely simulates the observations made in human and swine RVD, as it has been consistently noted that chronic RVD eventually reaches the same endpoints predicted by the Boolean model, including progressive loss of renal VEGF availability, persistent MV rarefaction, inflammation, and fibrosis^7,10^. A potential drawback of the inability to mimic a specific timescale is in comparing and analyzing the speed at which each variable in the model reaches steady state. This fact, in combination with the fact that each variable was only measured at either two or three timepoints during the referenced previous studies whereas the Boolean model can make predictions at any given point in time, complicate the ability to make insightful interpretations of the time it takes each biological variable to reach its end steady state. However, overall, the Boolean model is as accurate as possible and serves as a suitable framework for predicting treatment outcomes in RVD with the potential to be refined and, potentially, translated into other models with different timescales.

We are aware of the inability of the Boolean model to predict specific values for precise comparison to observed experimental values. That is, each variable can only be active/inactive or on/off at any given point in time. However, predictions made by Boolean models and oth er types of finite dynamical systems can often be verified experimentally more easily^4^, which may make this model advantageous over continuous mathematical models. For the purpose of our study, this does not represent a limitation since mechanistic studies were performed and the Boolean approach was employed as a mathematical tool to confirm significant biological findings in RVD and after treatments. Especially given the variability that may exist in human and swine RVD^7,34,37–40^, the ability to simultaneously analyze the states of multiple different variables involved in disease progression and make global-level predictions may be better suited for evaluating treatment outcomes in renal disease. Nonetheless, we recognize the potential value of quantitatively simulating RVD, and future work will aim to translate the network topology of MV rarefaction in RVD into a verified continuous mathematical model with the ability to predict specific values for each variable. Furthermore, in addition to working towards the Boolean model being able to predict specific values for comparison to experimental measurements, it will also be advantageous to work towards optimize the model for discrepancies that are identified between simulations and experimental measurements. These enhancements to the model will be considered in our future work.

There are other attractive components that encourage the application of the Boolean model into translational studies. This discrete model may allow us to identify any gaps or errors in our understanding of MV rarefaction and renal injury progression in RVD by comparing the simulations to our experimental studies. We^7,8,10,11,41^ and others^5,22–24,42^ have extensively studied the association of MV rarefaction with progression of renal injury in RVD and other forms of renal diseases, but precise physiological mechanisms underlying this disease and successful therapeutic strategies have not been fully elucidated. Thus far, Boolean simulations suggest that there is a vicious feed-forward cycle between oxidative stress and inflammation that may ultimately drive the progressive MV rarefaction, fibrosis, and renal injury and must be overcome to produce measurable renal recovery, making variables involved in oxidative stress and inflammation important key players in RVD pathophysiology. This is in line with our experimental observations, but the Boolean model supports the importance of these variables (and others) and may give us direction on which components should continue studying experimentally. In conjunction with our ongoing experimental studies, the use of the Boolean model to identify specific variables that may or may not play an integral role in the progression of RVD may contribute to our understanding of the disease pathophysiology and to unravel potential new therapeutic targets.

Finally, we developed a relatively simple but useful mathematical simulation that combines multiple processes that occur simultaneously in RVD and contribute to MV rarefaction for a more global view of the various pathophysiological pathways we showed to participate in the functional and structural deterioration of the stenotic kidney. Whereas experimentally we may be limited in the measurements able to take after targeted therapy, the model allows us to predict what is occurring with each and every variable in the network at steady state. Therefore, we can integrate different aspects of the pathophysiology of RVD that may not be able to dissect by performing experiments.

We recognize that our study shows some limitations. In this model, there are therapeutic strategies that have successfully protected the stenotic kidney *in vivo* but may not be suited to be discretely modeled. For example, we and others^9,29,43,44^ have demonstrated renoprotective effects of delivering endothelial progenitor cells to the stenotic kidney in RVD^9,29^. While cell-based therapies show promising results for renal recovery, studies are still underway to elucidate the underlying mechanisms of renoprotection and which factors involved in MV rarefaction are directly impacted by direct or cytokine-mediated actions of regenerative cells. However, with ongoing and future studies aimed to reach a more precise understanding of the mechanisms of cell-based therapy in renal disease, this therapeutic strategy may very well be able to be simulated by the model in the future.

In conclusion, the Boolean model was able to accurately predict *in vivo* and *ex vivo* experimental data and help to confirm the key role that MV rarefaction plays in the progression of renal injury. Furthermore, the Boolean model of MV rarefaction may be a useful tool for predicting outcomes of targeted treatments before performing time-intensiv e and costly experiments. Our study has unveiled a potentially useful tool for designing and indicating support or lack of support for pre-clinical experimental studies and supports potential for future use of Boolean networks to predict renal outcomes after targeted therapeutic strategies. While mathematical simulations cannot fully replace experimental studies, evaluation of predicted outcomes of therapeutic interventions may be very useful in designing treatments that are supported by this verified disease simulation before *in vivo* testing begins. Future studies will determine whether this discrete model can be successfully mirrored by a more quantitative continuous mathematical model and, if so, may determine if a more complex model will be of potential use as the Boolean model is.

## Methods

A network topology was created of 19 factors known to be involved in MV rarefaction and the progression of renal injury in RVD.

### Description and supporting references of variables in the model and their involvement in MV rarefaction and progression of renal injury in RVD

#### 1 = Chronic blood flow (BF)

Total renal blood flow to the kidney^7,8,12,13,20,35^. Values are normal in the healthy, unobstructed kidney, but significantly decreased in renal artery stenosis and RVD^7,8,12,13,20,35^.

#### 2 = Mild renal ischemia

Reduced tissue (renal) oxygen content^25,45–47^. Acute and chronic reductions in blood flow decrease oxygen availability in the stenotic kidney, which is a major stimulus for the release and activation of many factors involved in inflammation, oxidative stress, and MV rarefaction^25,45–47^.

#### 3 = NADPH oxidase

Source of free radicals^48^. Activation of NADPH oxidases greatly contributes to the production of superoxide, which may generate reactive oxygen species and increase oxidative stress in the stenotic kidney^48^.

#### 4 = Ang II/ET-1, denoted as ‘Ang II’

Vasoconstrictors^49^. Hypoxia in the stenotic kidney is a major stimulus for Ang II and other vasoconstrictors, which increase vasoconstriction and play a role in the increase in oxidative stress and inflammation that occurs in RVD^49^.

#### 5 = ROS scavenging

Scavenging of reactive oxygen species to reduce oxidative stress^50^. The number of scavengers of free radicals and other reactive oxygen spec ies is decreased in chronic ischemia, as occurs in RVD^50^.

#### 6 = Ox-LDL

Free radical oxidized low-density lipoprotein^51^. Free radicals resulting from increased oxidative stress reacting with low-density lipoprotein have the propensity to activate and perpetuate inflammatory processes in the diseased kidney^51^.

#### 7 = ROS/Ox stress

Increased production of reactive oxygen species. Increased oxidative stress has been demonstrated in RVD and other renal diseases and likely plays an important role in the pathogenesis of renal injury^35,52^.

#### 8 = NFkB

Induces transcription of pro-inflammatory cytokines^53–55^. NFkB mediates the transcription of multiple pro-inflammatory cytokines and has been linked to several renal diseases^53–55^.

#### 9 = Nitric oxide (NO)

Vasodilator^56–59^. Deficient nitric oxide release often reflects endothelial dysfunction and occurs in renal diseases in which endothelial dysfunction is present and develops early in the disease^56–59^.

#### 10 = VEGF

Pro-angiogenic cytokine, maintains the integrity of microvessels^7,8,10^. Renal MV rarefaction that occurs during the progression of RVD associates with a decrease in bioavailability of endogenous VEGF^7,8,10^.

#### 11 = Angiostatin/Endostatin/Thrombospondin, denoted as ‘Angiostatin’

Anti-angiogenic cytokine^60^. Angiostatin and similar factors are elevated in ischemic renal injury and reduce the effects of VE GF and its downst ream mediators^60^.

#### 12 = Vasoconstriction

The endothelial dysfunction that occurs in the ischemic kidney along with the pro-inflammatory, anti-angiogenic environment may contribute to increased intra-renal vasoconstriction.

#### 13 = MV rarefaction

Reduction in MV density^5,6,61,62^. MV abnormalities, including rarefaction and regression, are prominent features in chronic renal disease irrespective of the initial cause^5,6^.

#### 14 = Inflammation

Evidence of renal inflammation in RVD has been consistently demonstrated^35,52^.

#### 15 = TGF-beta

Pro-fibrotic, pro-inflammatory cytokine^14^. TGF-beta and other pro-fibrotic, pro-inflammatory factors have been demonstrated to be elevated in in RVD^14^.

#### 16 = Glomerulosclerosis (Sclerosis)

Glomerular scarring^63^. The RVD kidney has been shown to have significantly increased glomerulosclerosis compared to normal^63^.

#### 17 = TIMP/MMPs

Balance between matrix metallopeptidases and their inhibitors^64^. TIMPs and MMPs are several important regulators of extracellular matrix turnover in the kidney, and a shift in the ratio or balance between these two antagonistic proteins can impact tissue remodeling^64^.

#### 18 =  More severe renal ischemia

Lack of oxygen disrupting cellular metabolism^65^. Ischemic nephropathy occu rs when renal blood flow is obstructed and compromises the kidney's ability to excrete properly, which often occurs in RVD^65^.

#### 19 = Tubule-interstitial injury and fibrosis (Fibrosis)

Accumulation of interstitial collagen^8,13,18,20,31^. The RVD kidney has been shown to have significantly increased tubule-interstitial fibrosis compared to normal^8,13,18,20,31^.

### Description of variables simulating therapeutic strategies in the model

Variables simulating different therapeutics and interventions used to compare the model against previously published data were also included as variables in the Boolean model:

#### 20 = VEGF administration

simulates restoration of VEGF in the stenotic kidney by activating the endogenous VEGF variable in the model^7,8,12,13,20^.

#### 21 = Simvastatin

simulates Simvastatin therapy which targets variables associated with oxidative stress and inflammation and inactivates them in the model^14^.

#### 22 = Renal artery stenosis (RAS)

simulates renal artery stenosis by inactivating renal blood flow in the model^66^. When blood flow is re-activated, the model simulates renal angioplasty.

#### 23 = Anti-oxidant administration (Vitamins C and E)

simulates therapy with anti-oxidants by targeting and inactivating oxidative stress in the model^15–17^.

#### 24 = Endothelin-A (ET_A_) receptor blockade

simulates antagonism of the Endothelin-A (ET_A_) receptor by inactivating ET-1/Ang II in the model^19^.

### Boolean model functions

Based on the network topology of MV rarefaction and renal injury progression in RVD, the status of each variable at any given timepoint was assumed to be either “on” or “off”. Functions determining the next state of a variable based on its interaction with other variables in the framework were defined in terms of the Boolean operators ∧ and ∨ (logical AND and OR). The values 0 and 1 are the states of the variables, with 0 representing “off” and 1 representing “on”.

In the following functions, the operator ∧ indicates that both variables influencing the variable of interest need to be present, or activated, in order to synergistically activate the variable of interest. Alternatively, the operator ∨ indicates that either influencing variable's presence or activation is sufficient for activation for the variable of interest to occur, and the influencing variables act independently of one another. The operator ¬ indicates that the variable described is a repressor and must be absent or inactive for activation of the variable of interest to occur. The Round operator is used to round the real number outcome to an integer (0 or 1) in the event that the effect of multiple variables on the activity of the variable of interest comes out to a value that is in between 0 and 1. Interactions that maintain the variable of interest (a) in its current state, whether active or inactive, are denoted by a direct relationship with the influencing variable (b): F_*a*_ = *b*, whereas interactions that cause a switch in the state of the variable of interest (a) are defined by the “rule”: F_*a*_ = ¬*b*. For any variable *a*, the function described as F_*a*_ determines the activity or inactivity of *a* after one unit of time. The Boolean function for each variable in the model listed above are as follows (see Fig. 1 for an illustration of relationships defined by Boolean functions). For clarity, the relationship between variables described mathematically are also described in written form:

#### Boolean function for 1

F_1_ = $\neg \,$22 ∧ $\neg \,$13. For chronic blood flow in the kidney to be on or active, renal artery stenosis and MV rarefaction must be absent.

#### Boolean function for 2

F_2_ = $\neg \,$1. Mild renal ischemia is only present when chronic blood flow is absent or turned off.

#### Boolean function for 3

F_3_ = 2 ∧ 4. NADPH oxidase is active when mild ischemia and angiotensin II/Endothelin-1 are active.

#### Boolean function for 4

F_4_ = ¬24 ∧ (2 ∨ 18). Ang II/ET-1 vasoconstrictors are active when an ET_A_ receptor blocker is not currently being administered and when mild ischemia or tissue ischemia are present/active.

#### Boolean function for 5

F_5_ = 21 ∨ ¬3. ROS scavenging is active when exogenous statins are administered or when NADPH oxidase is inactive.

#### Boolean function for 6

F_6_ = $\neg \,$21 ∧ ($\neg \,$5 ∨ 3 ∨ 8). Ox-LDL is active when exogenous statins are not being administered and ROS scavenging is inactive or NADPH oxidase or NFkB are active.

#### Boolean function for 7

F_7_ = ¬23 ∧ (¬5 ∨ 15 ∨ ¬9). ROS/Oxidative stress is active when exogenous anti-oxidants are not being administered and ROS scavenging or nitric oxide are inactive or NADPH oxidase is active.

#### Boolean function for 8

F_8_ = ¬21 ∧ (6 ∧ 18). NFkB is active if simvastatin is not being administered and Ox-LDL and tissue ischemia are active.

#### Boolean function for 9

F_9_ = ¬7 ∨ 10 ∨ 21. Nitric oxide is active if ROS/oxidative stress is inactive or VEGF is active or simvastatin is administered.

#### Boolean function for 10

F_10_ = ¬7 ∨ 20. VEGF is active when ROS/oxidative stress is absent/inactive or exogenous VEGF is administered.

#### Boolean function for 11

F_11_ = 7. Activation of angiostatin requires ROS/oxidative stress to be present/active.

#### Boolean function for 12

F_12_ = Round (Mean 4, ¬9, 14). Vasoconstriction occurs when the mean outcome of Ang II/ET-1 activity, inactivity of nitric oxide, and activity of inflammation indicates presence/activity (value of 1) when rounded.

#### Boolean function for 13

F_13_ = 11 ∧ 12. MV rarefaction is active when vasoconstriction and angiostatin are both present/active.

#### Boolean function for 14

F_15_ = 8 ∨ 4. Activation of inflammation requires either NFkB or angiotensin II/endothelin-1 to be present/active.

#### Boolean function for 15

F_16_ = 14. TGF-beta is active only if inflammation is active.

#### Boolean function for 16

F_17_ = 17. Glomerulosclerosis is present/active if the balance between TIMP-1 and MMPs favors the activity of TIMP-1.

#### Boolean function for 17

F_18_ = Round ((4 + 7 + 15)/3). TIMP-1 activity becomes more active than its antagonistic MMP activity if the rounded average between Ang II/ET-1, ROS/oxidative stress, and TGF-beta is active.

#### Boolean function for 18

F_19_ = 13. More severe renal ischemia is active if MV rarefaction is also active.

#### Boolean function for 19

F_20_ = 16. Fibrosis is active when glomerulosclerosis is also active.

#### Boolean function for 20

F_21_ = 20. VEGF administration is activates VEGF when simulated.

#### Boolean function for 21

F_22_ = 21. Simvastatin administration affects ROS scavenging, Ox-LDL, and NFkB when simulated.

#### Boolean function for 22

F_23_ = 22. Renal artery stenosis is affects chronic blood flow when simulated.

#### Boolean function for 23

F_24_ = 23. Anti-oxidant administration (Vitamins C and E) affects ROS/oxidative stress when simulated.

#### Boolean function for 24

F_25_ = 24. ET_A_ receptor blockade affects Ang II/ET-1 when simulated.

### Description of initial state for each simulation

For each simulation, the model is given an initial state in which each variable is assigned to be either active or inactive (0 or 1) at the beginning of the run. The initial state of the model reflects a kidney in a quiescent state, with no deleterious disease processes activated. (See the first column of Figs. 2–8 for a visual representation of the initial state of each simulation).

RVD with no intervention: (1, 0, 0, 0, 1, 0, 0, 0, 1, 1, 0, 0, 0, 0, 0, 0, 0, 0, 0, 1, 0, 0)

RVD with Simvastatin administration: (1, 0, 0, 0, 1, 0, 0, 0, 1, 1, 0, 0, 0, 0, 0, 0, 0, 0, 1, 1, 0, 0)

RVD with Anti-oxidant (Vitamins C and E) administration: (1, 0, 0, 0, 1, 0, 0, 0, 1, 1, 0, 0, 0, 0, 0, 0, 0, 0, 0, 1, 1, 0)

RVD with ET_A_ receptor blockade therapy: (1, 0, 0, 0, 1, 0, 0, 0, 1, 1, 0, 0, 0, 0, 0, 0, 0, 0, 0, 1, 0, 1)

RVD with VEGF administration at disease onset intervention (preventative): (1, 0, 0, 0, 1, 0, 0, 0, 1, 1, 0, 0, 0, 0, 0, 0, 0, 1, 0, 1, 0, 0)

RVD with VEGF administration after disease progression intervention: (1, 0, 0, 0, 1, 0, 0, 0, 1, 1, 0, 0, 0, 0, 0, 0, 0, 0, 0, 1, 0, 0), with a switch at timepoint 25 to (1, 0, 0, 0, 1, 0, 0, 0, 1, 1, 0, 0, 0, 0, 0, 0, 0, 1, 0, 1, 0, 0)

RVD with combined angioplasty and VEGF intervention: (1, 0, 0, 0, 1, 0, 0, 0, 1, 1, 0, 0, 0, 0, 0, 0, 0, 0, 0, 1, 0, 0), with a switch at timepoint 25 to (1, 0, 0, 0, 1, 0, 0, 0, 1, 1, 0, 0, 0, 0, 0, 0, 0, 1, 0, 0, 0, 0)

The initial states and algorithms determining relationships amongst variables were set up based on the current understanding of the pathophysiology of RVD prior to running each therapeutic simulation. There was no calibration phase to optimize the Boolean model prior to running simulations to determine end steady states.

## Data Availability

All data generated or analyzed during this study are included in this article. All algorithmic code used for this study is included in this article. Code is available upon request.
